# Latent profile analysis of cognitive decline and depressive symptoms after intracerebral hemorrhage

**DOI:** 10.1186/s12883-021-02508-x

**Published:** 2021-12-10

**Authors:** Sophia Keins, Jessica R. Abramson, Juan Pablo Castello, Marco Pasi, Andreas Charidimou, Christina Kourkoulis, Zora DiPucchio, Kristin Schwab, Christopher D. Anderson, M. Edip Gurol, Steven M. Greenberg, Jonathan Rosand, Anand Viswanathan, Alessandro Biffi

**Affiliations:** 1 Department of Neurology, Massachusetts General Hospital, 100 Cambridge Street - Room 2064, Boston, MA 02114 USA; 2 Hemorrhagic Stroke Research Program, Massachusetts General Hospital, Boston, MA USA; 3 Henry and Allison McCance Center for Brain Health, Massachusetts General Hospital, Boston, MA USA; 4 Department of Psychiatry, Massachusetts General Hospital, Boston, MA USA; 5 Center for Genomic Medicine, Massachusetts General Hospital, Boston, MA USA; 6 Univ.Lille, Inserm, CHU Lille, U 1172 - LilNCog - Lille Neuroscience and Cognition, F-59000 Lille, France

**Keywords:** Intracerebral hemorrhage, Neuropsychiatric outcomes, Cerebral small vessel disease, Memory disorders, Mood disorders

## Abstract

**Background:**

Cognitive impairment and depressive symptoms are highly prevalent after Intracerebral Hemorrhage (ICH). We leveraged Latent Profile Analysis (LPA) to identify profiles for cognitive decline and depression onset after ICH. We also investigated differences in clinical, genetic and neuroimaging characteristics across patients’ profiles.

**Methods:**

We analyzed data from the ICH study conducted at Massachusetts General Hospital between January 1998 and December 2019. We collected information from electronical health records, follow-up interviews, CT and MRI imaging, and *APOE* genotype. We conducted LPA and multinomial logistic regression analyses to: 1) identify distinct profiles for cognitive decline and depression onset after ICH; 2) identify clinical, neuroimaging and genetic factors predicting individuals’ likelihood to express a specific profile.

**Results:**

We followed 784 ICH survivors for a median of 45.8 months. We identified four distinct profiles in cognitive and depressive symptoms after ICH: low depression and dementia risk, early-onset depression and dementia, late-onset depression and dementia, high depression with low dementia risk. Cerebral small vessel disease severity and *APOE* genotype were specifically associated with the late-onset profile (both *p <* 0.05). Acute hematoma characteristics (size, intraventricular extension) and functional disability were specifically associated with the early-onset profile (all *p <* 0.05).

**Conclusion:**

We identified four distinct profiles for cognitive and depressive symptoms after ICH, each displaying specific associations with individual patients’ clinical, genetic and neuroimaging data. These associations reflect separate biological mechanisms influencing dementia and depression risk after ICH. Our findings support employing LPA in future ICH studies, and is likely applicable to stroke survivors at large.

**Supplementary Information:**

The online version contains supplementary material available at 10.1186/s12883-021-02508-x.

## Introduction

Survivors of Intracerebral Hemorrhage (ICH) are at high risk for cognitive decline following the acute hemorrhage event, with up to 40% of patients developing dementia within 5 years [1]. Between one third and half of all ICH survivors also develop post-stroke depression within 5 years of the initial hemorrhagic stroke [2, 3]. Cognitive decline and depression onset are both associated with poor long-term functional outcomes following stroke in general, and ICH in particular [4–8]. Multiple studies, including from our group, also clarified that cognitive decline and depressive symptoms often overlap among ICH survivors [3, 9].

Cognitive decline and depression onset after stroke are both considered the final manifestation of a complex network of genetic, societal / environmental, and individual factors [4, 10]. Patient’s demographics and social determinants of health are established predictors of neurocognitive and neuropsychiatric outcomes after stroke [11, 12]. History of cognitive impairment or depression before stroke have also been repeatedly associated with higher risk for dementia and persistent depressive symptoms after stroke [5]. Acute hemorrhage characteristics (e.g. size, anatomical location) are also associated with cognitive decline and depression risk after hemorrhagic stroke [8, 10]. In addition, most primary ICH events represent an acute manifestation of underlying cerebral small vessel disease (CSVD), a progressive degenerative disorder of small calibers arterioles in the central nervous system [7]. CSVD is associated with increased risk for depression and cognitive decline, both in the general population and among ICH survivors [5, 13].

However, the relationship between cognitive decline and depressive symptoms following ICH remains only partially understood, both in terms of clinical manifestations and underlying biological mechanisms [1, 4]. Improved understanding of the biological mechanisms influencing risk for cognitive decline and depression after ICH (including how they jointly affect clinical outcomes) would greatly accelerate development of novel, targeted treatments for current and future survivors [2, 8]. More accurate prognostication of future risk for cognitive decline and depression would also better assist clinicians caring for ICH survivors [14].

Therefore, we leveraged data from the longitudinal study of ICH survivors conducted at Massachusetts General Hospital (MGH-ICH study) to examine the relationship between depression onset and cognitive decline after ICH. We specifically sought to identify distinct profiles for cognitive decline and depression over time after ICH. To this end, we employed an unbiased bioinformatic approach in the form of Latent Profile Analysis (LPA). LPA (and other related methods in the framework of latent class analysis) allow investigators to identify patient subgroups sharing similarities in clinical disease manifestations [15]. LPA and similar methods have been recently employed to identify subtypes of major depression and characterize late-life cognitive trajectories [16, 17]. We also surveyed information on patients’ demographics, clinical history, genetics, acute ICH characteristics and underlying CSVD to identify predictors of specific profiles for depression and cognitive decline after ICH.

## Methods

### Study participants and eligibility criteria

We analyzed data for ICH survivors consecutively enrolled in the single-center Massachusetts General Hospital ICH (MGH)-ICH study [9, 18]. Participants were adults (age ≥ 18), presenting between January 1998 and December 2019 and diagnosed with primary (i.e. spontaneous) ICH. All ICH diagnoses were confirmed on CT scan obtained within 24 h of symptoms’ onset. Individuals presenting with secondary ICH (i.e. due to trauma, transformation of ischemic infarct, infection, demyelinating lesion, intracranial tumor, ruptured aneurysm, or other vascular abnormalities) were ineligible. Because we sought to identify evolution in cognitive symptoms over time after ICH, we excluded participants with pre-ICH dementia (since they had already and irreversibly developed our outcome of interest). We defined pre-ICH dementia as meeting either of the following criteria: 1) history of dementia (by DSM-5 criteria) based on review of medical records and ICD-9 / ICD-10 based billing codes; or 2) score > 3.3 on the 16-item (short) version of the Informant Questionnaire on Cognitive Decline in the Elderly (IQCODE) [1, 5]. In contrast, previous studies support the hypothesis that a pre-stroke diagnosis of depression does not inevitably result in the development of post-stroke depression, especially if no depressive symptoms are present immediately after stroke [11]. We therefore opted not to exclude individuals with history of pre-ICH depression or other mood disorders, defined as meeting both the following criteria: 1) self-reported or informant-reported prior diagnosis of any mood disorder; 2) history of any mood disorders (by DSM-5 criteria) based on review of medical records and ICD-9 / ICD-10 based billing codes [9].

### Participants’ enrollment and baseline data collection

Eligible individuals (or their surrogates) underwent a structured research interview to collect demographic and medical history data, supplemented via semi-automated review of medical records and billing codes [9, 19]. We determined whether participants had pre-ICH history of depression or other mood disorders, We determined *APOE* genotype on DNA extracted from blood samples, as previously described [20]. Research staff in charge of enrollment and capture of baseline clinical information were blinded to neuroimaging, genetic and follow-up data.

### Longitudinal follow-up

We contacted participants and/or their surrogates by phone every 3 months after index ICH for the first year, and every 6 months thereafter [19]. We gathered information and reviewed medical records pertaining to recurrent stroke, death, and changes in medication regimens. We also administered the following scales: 1) modified Rankin Scale (mRS); 2) the Katz and Lawton questionnaires for Activities of Daily Living (ADLs) and Instrumental Activities of Daily Living (IADLs) [21, 22]; 3) the 4-item version of the Geriatric Depression Scale (GDS-4) to determine presence vs. absence of active depressive symptoms [23]; 4) the modified Telephone Interview for Cognitive Status (TICS-m), a validated telephone-based, global cognitive assessment tool that measures overall cognitive performance - with scores ranging from 0 (worst performance) to 41 (best performance) [24–26]. Study staff augmented phone-based follow-up data with semi-automated review of longitudinal electronic medical records [9]. We specifically extracted data on use of antidepressant medications, based on previously described methods [3].

### Neuroimaging data capture and analysis

We determined hematoma location and presence of Intraventricular Hemorrhage (IVH) on arrival CT scans, based on consensus review by study staff [19]. ICH location was defined as lobar (selective involvement of the cortex and/or subcortical white matter), non-lobar (selective involvement of thalamus, basal ganglia, cerebellum or brainstem) or multiple locations. We used an established semi-automated software algorithm to determine ICH volume [9]. MRI Images were obtained using a 1.5 or 3.0 Tesla MR scanner (GE Sigma), using a previously validated methodology (see Supplemental Material) [27]. Neuroimaging markers of CSVD severity were rated according to STRIVE consensus criteria, as previously described [27, 28]. Based on a recently described and validated total CSVD score, we rated global CSVD burden on an ordinal scale from 0 to 6 [29]. We allocated one point for presence of: a) lacunes; b) 1–4 cerebral microbleeds (CMBs); c) moderate to severe basal ganglia Expanded Peri-Vascular Spaces (EPVS), i.e. count > 20; d) moderate White Matter Hyperintensities (WMH), i.e. total periventricular + subcortical WMH grade 3–4. We allocated 2 points for presence of: a) ≥ 5 CMBs; and b) severe WMH, i.e. total periventricular + deep WMHs grade 5–6. All ICH patients were classified based on the location of ICH and CMBs, and presence of Cortical Superficial Siderosis (CSS) as Hypertensive Arteriopathy (HTNA)-related ICH, Cerebral Amyloid Angiopathy (CAA)-related ICH and mixed-ICH (See Supplemental Material) [30].

### Exposures and outcomes of interest

Age at index ICH was analyzed as a continuous variable. Race/ethnicity was analyzed as a categorical variable, with white patients as the reference group owing to their numerical preponderance. Education level was dichotomized using ≥12 years as the cutoff. Lobar vs. non-lobar ICH location was analyzed as a dichotomous variable. ICH volume was analyzed as a continuous variable. We analyzed mRS as an ordinal variable. We used two separate ordinal variables capturing scores for ADLs and IADLs questionnaires. CSVD MRI markers were analyzed as discussed above. Scores from the TICS-m and GDS-4 underwent z-score transformation for all subsequent analyses. We defined incident dementia for outcome analyses as patients meeting both of the following criteria: 1) relevant ICD-9 or ICD-10 codes entered in electronic medical records; and 2) diagnosis confirmed by semi-automated review of medical records, as previously described [27]. We defined depression for outcome analyses as patients meeting both of the following criteria: 1) relevant ICD-9 or ICD-10 codes entered in electronic medical records; and 2) diagnosis confirmed by semi-automated review of medical records, as previously described [3].

### Statistical methods

Categorical variables were compared using Fisher exact test (two-sided) and continuous variables using the Mann-Whitney rank-sum or unpaired t test. In order to identify cognitive decline and depression onset profiles after ICH we utilized Latent Profile Analysis (LPA) [15]. We utilized scores for the GDS-4 and TICS-m at each follow-up interval (i.e. at 3 months after ICH, at 6 months after ICH, and every 6 months thereafter) as input for LPA. Missing information due to death or loss to follow-up was handled by carrying forward the last available value. We then used this time-dependent information to identify latent profiles corresponding to different temporal patterns in appearance of cognitive and depressive symptoms after ICH. We utilized the Bayesian Information Criterion (BIC) criterion to identify the optimal number of profiles. We then performed univariable and multivariable analyses (multinomial logistic regression) to identify predictors of patients’ assignment to latent categories of interest. Candidate predictors included demographics, medical history, CT and MRI information, and APOE genotype. All variables with *p* < 0.20 for univariable association with latent profiles assignment were included in multivariable analyses. After variable selection, we generated minimal models by backward elimination of non-significant variables (*p* > 0.05). Multicollinearity was assessed by computing Variance Inflation Factors (VIF) for all predictors and removing all variables with VIF > 5 (none required removal as part of the analyses presented in the Results section). We adjusted for multiple testing burden (including all univariable analyses and the multinomial logistic regression results) using the False Discovery Rate (FDR) method, and significance was set at *p* < 0.05 (after FDR adjustment) [31]. All analyses were performed using the STATA software (StataCorp), v16.0.

### Data availability

The authors certify they have documented all data, methods, and materials used to conduct the research presented. Anonymized data pertaining to the research presented will be made available by the corresponding author, upon reasonable request from external investigators.

## Results

### Study participants and follow-up information

**Fig. 1:**
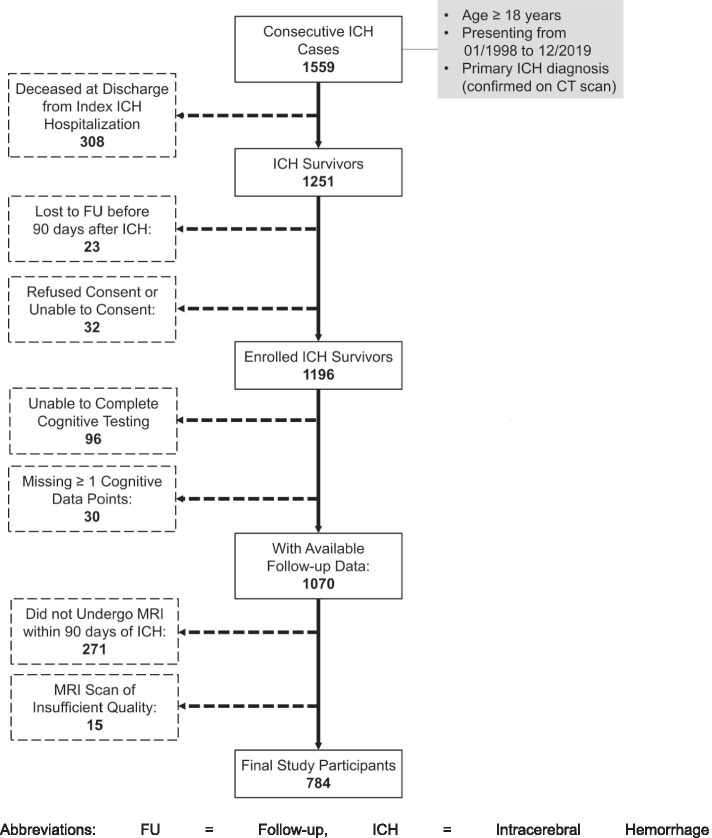
Study Inclusion and Exclusion Criteria. Abbreviations: FU = Follow-up, ICH = Intracerebral Hemorrhage

After application of pre-specified inclusion and exclusion criteria to 1559 consecutive ICH cases (Fig. 1), we analyzed data for 784 survivors of primary ICH enrolled in our longitudinal study. Mortality at time of discharge from index ICH hospitalization was the most common reason for exclusion, accounting for 308 of 775 excluded cases (40%). Lack of MRI data of sufficient quality for analysis was the second most common reason for exclusion, affecting 286 participants (37% of all excluded participants). We found no significant differences in demographics, medical history, or ICH location and volume when comparing ICH survivors with vs. without MRI data (all *p* > 0.20). For ICH survivors with available MRI data, median time from hospital arrival to scan was 4.2 days (Inter-Quartile Range [IQR]: 3.3–5.8). Participants were followed for a median time of 45.8 months (Inter-Quartile Range [IQR]: 35.3–59.2). We estimated average yearly loss to follow-up at 1.2%. We present key characteristics for participating ICH survivors in Table 1. During follow-up a total of 266/784(34%) study participants met our pre-specified diagnostic criteria for dementia. In addition, a total of 383/784 (49%) study participants met diagnostic criteria for depression during follow-up.

**Table 1:** Characteristics of Study Participants

Variable	No.	%
**No. of Individuals**	**784**	**100**
**Demographics**		
Age at Enrollment (Mean, SD)	70.9 (12.8)	–
Sex (Male)	408	52
Race / Ethnicity	0	
- White	659	84
- Hispanic	55	7
- Black	47	6
- Other	23	3
Education (≥12 years)	463	59
**Medical History**		
Hypertension	596	76
Diabetes	157	20
Pre-ICH Depression	133	17
Coronary Artery Disease	118	15
Atrial Fibrillation	118	15
Previous Functional Dependence	71	9
Prior Ischemic Stroke / TIA	63	8
Prior ICH	24	3
**Acute ICH Hospitalization**		
Admission GCS (median, IQR)	14 (9–15)	–
Discharge mRS (median, IQR)	4 (3–5)	–
**CT Imaging Data**	0	
ICH Location	0	
- Lobar	416	53
- Non-lobar	353	45
- Mixed locations	15	2
ICH Volume (cc, median and IQR)	18.5 (6.1–29.8)	–
Intraventricular Extension	204	26
**MRI Data**		
ICH Etiological Classification		
- CAA-related ICH	400	51
- Mixed ICH	196	25
- HTNA-related ICH	188	24
Global CSVD Score (median, IQR)	2 (1–3)	–
**Genetic Data**		
APOE ε4 (≥ 1 copy)	165	21
APOE ε2 (≥ 1 copy)	110	14
**Variable**	**No.**	**%**
**No. of Individuals**	**784**	**100**
**Medication Use after ICH**		
Statins	274	35
SSRI	172	22
Antiplatelet Agents	125	16
Oral Anticoagulants	78	10

All values presented as number and percentage, unless otherwise specified

Abbreviations: *CAA* Cerebral Amyloid Angiopathy, *CSVD* Cerebral Small Vessel Disease, *GCS* Glasgow Coma Scale, *HTNA* Hypertensive Arteriopathy, *ICH* Intracerebral Hemorrhage, *IQR* Inter-quartile Range, *mRS* modified Rankin Scale, *SD* Standard Deviation, *SSRI* Selective Serotonin Reuptake Inhibitor

### Identifying cognitive decline and depression onset profile after ICH

**Fig. 2:**
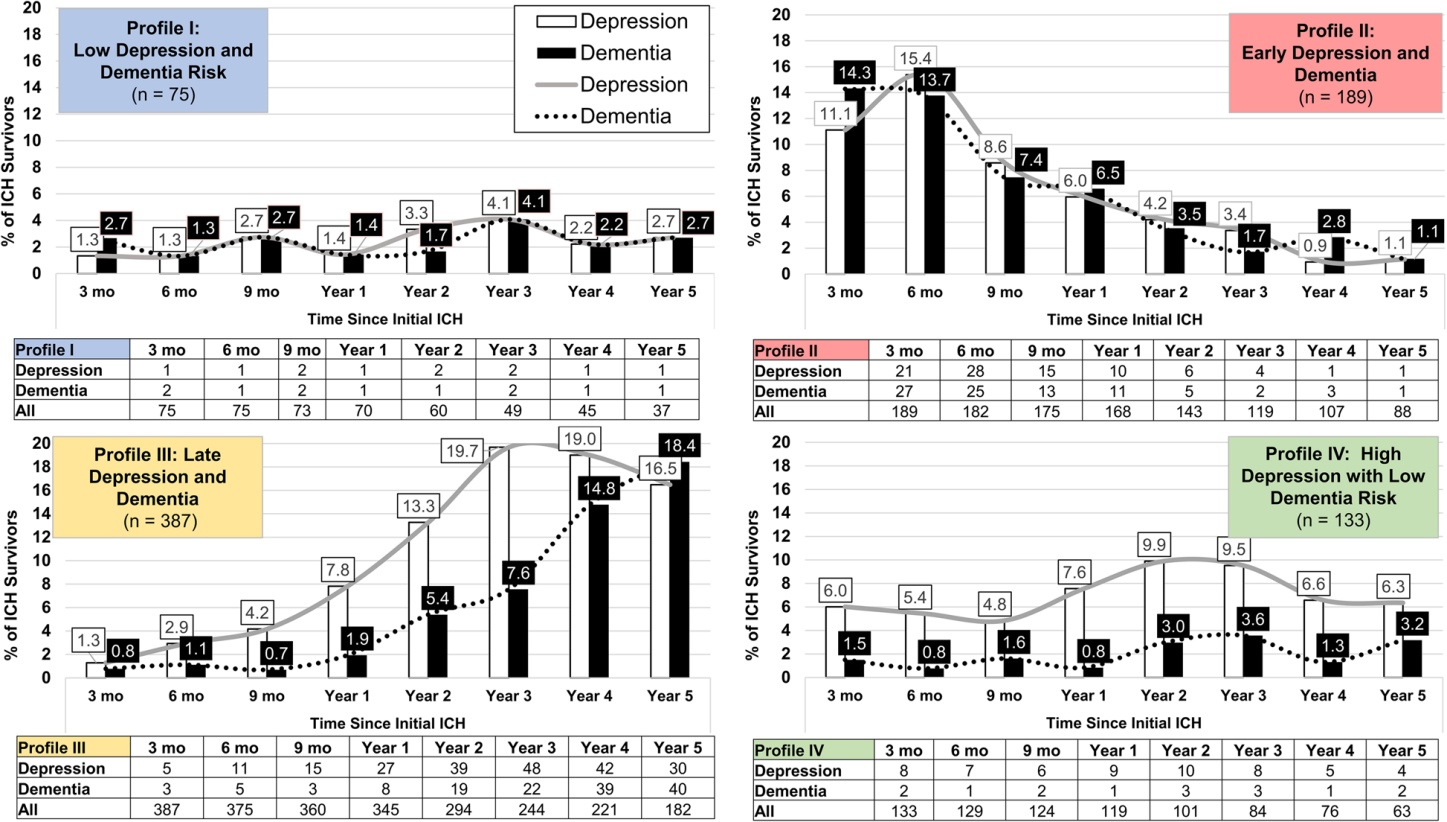
Cognitive Decline and Depression Onset Profiles after ICH. Each panel presents information on incidence of dementia and depression within patient subgroups defined by specific profiles in cognitive and mood symptoms after ICH: 1) Profile I (top left), are patients at low risk for both dementia and depression during follow-up after ICH; 2) Profile II (top right), are patients at high risk for both dementia and depression early in follow-up after ICH; 3) Profile III (bottom left), are patients at high risk for both dementia and depression later in follow-up after ICH; 4) Profile IV (bottom right), are patients at high risk for depression, but not dementia, during follow-up after ICH. For each profile, we report the percentage of ICH survivors developing dementia (black bars) and depression (white bars). Labels above each bar indicate the exact percentage of participants developing dementia or depression at each time point. Dementia (dotted black line) and depression (solid grey line) incidence over time are also presented for each profile. We report number of patients in follow-up and new depression / dementia diagnoses for each time point during follow-up in the tables below each figure. Abbreviations: ICH = Intracerebral Hemorrhage

We performed LPA analysis on testing scores for cognitive function (TICS-m) and depressive symptoms (GDS-4) over time. Based on the BIC criterion a total of four profiles represented the best fit model for data provided. For each study participants, the LPA model generated individual, patient-specific probabilities of assignment to each of the four profiles. Individual ICH survivors were then assigned to the profile with the highest probability for all subsequent analyses. We present the incidence of depression and dementia among participants, subdivided by profile, in Fig. 2. Based on the incidence of dementia and depression over time, we designated profiles as follows: Profile I as “low depression and dementia risk” (*n* = 75), Profile II as “early-onset depression and dementia” (*n* = 189), Profile III as “late-onset depression and dementia” (*n* = 387), Profile IV as “high depression with low dementia risk” (*n* = 133).

### Determinants of cognitive decline and depression onset profiles after ICH

**Fig. 3:**
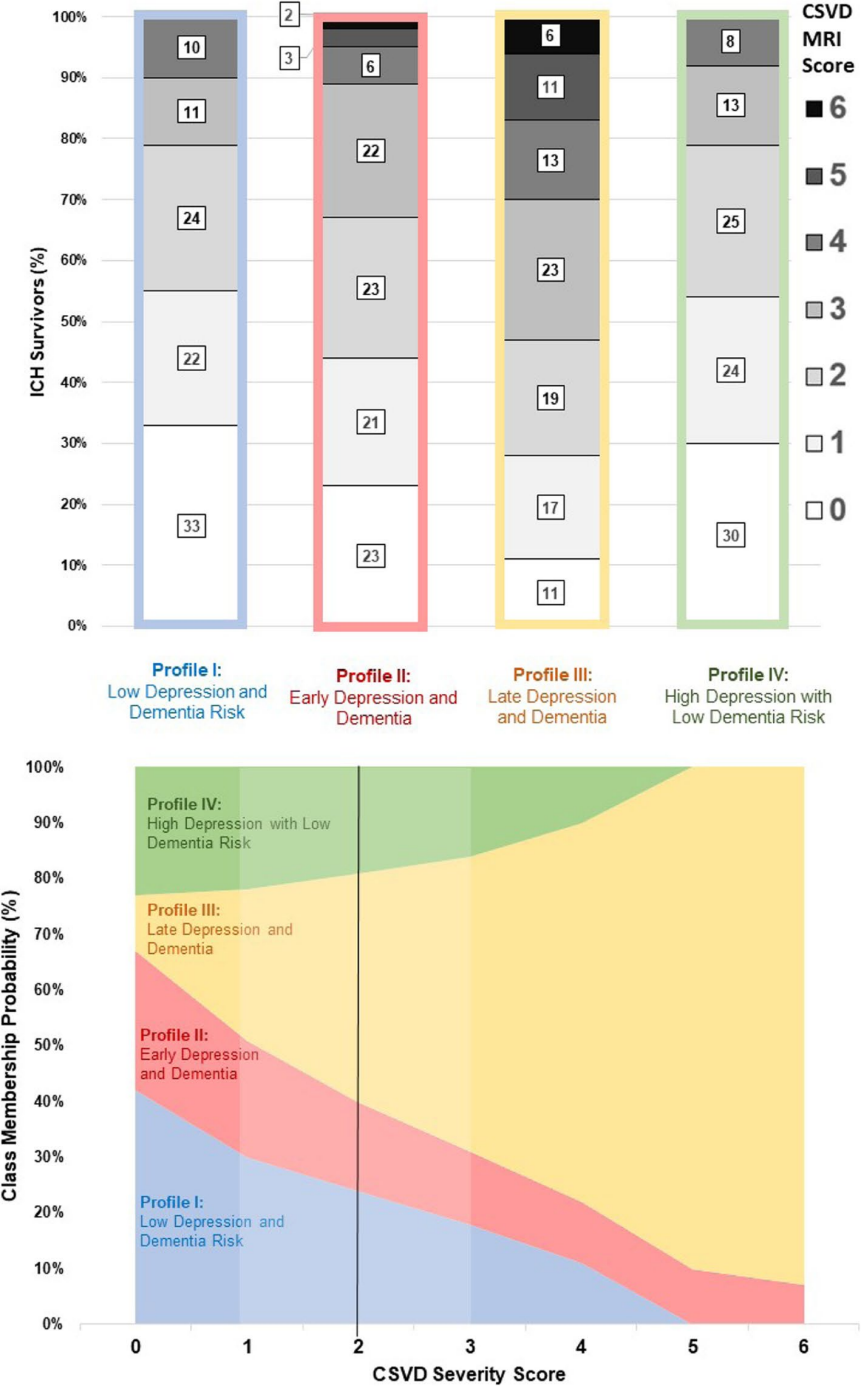
Cerebral Small Vessel Disease Severity and Cognitive Decline / Depression Onset Profiles after ICH. Association between CSVD severity score and patient subgroups identified by specific profies in cognitive and mood symptoms after ICH. Top panel: bar graph presenting CSVD severity (percentage of patients in each score category) for each profile subgroup. Bottom Panel: area graph presenting the relationship between increasing CSVD severity score and individual participants’ probability of expressing specific profiles. The solid vertical line indicates the median CSVD score value among study participants, while the shaded area encompasses the interquartile range. Abbreviations: CSVD = Cerebral Small Vessel Disease, ICH = Intracerebral Hemorrhage

**Fig. 4:**
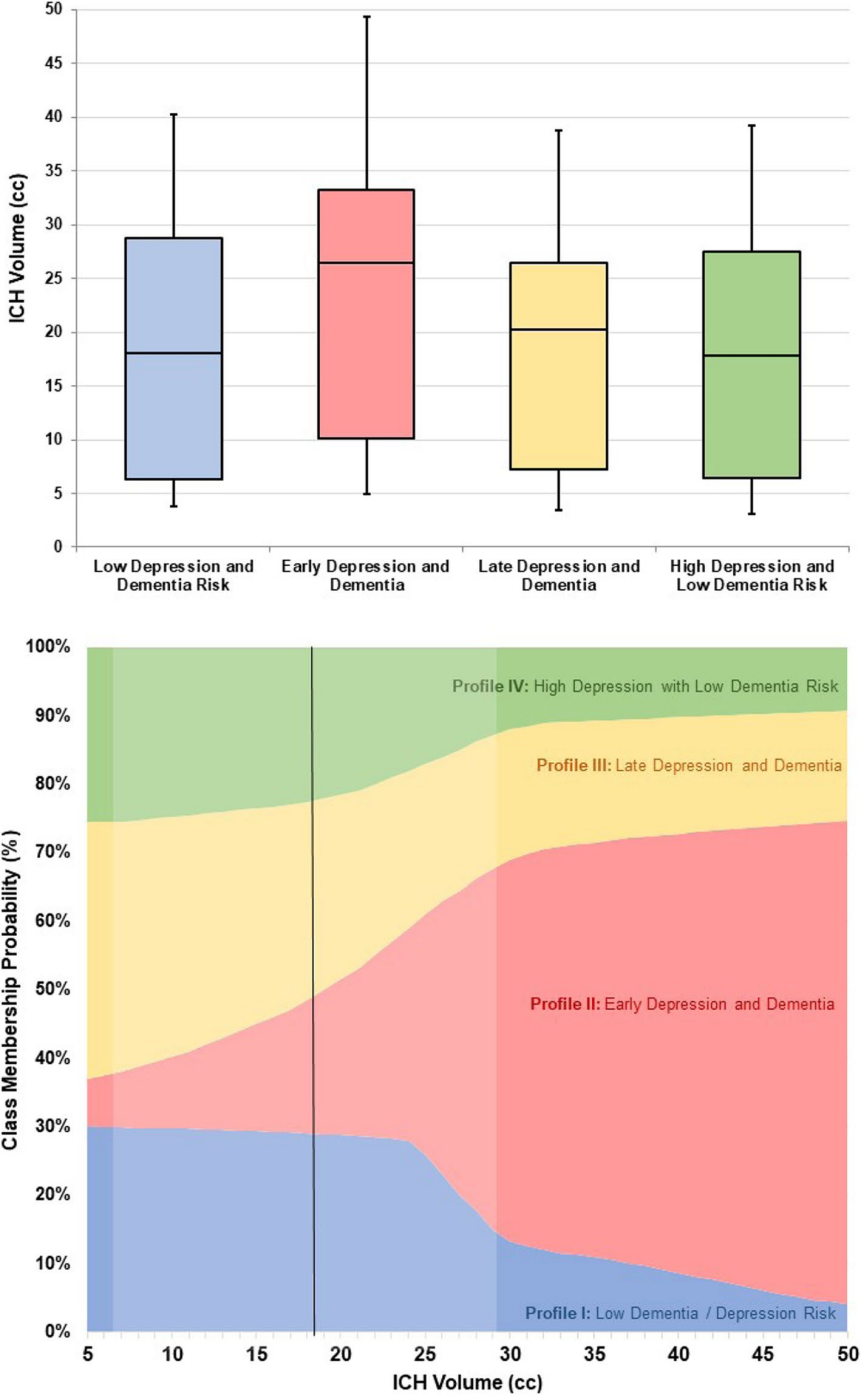
Hematoma Volume and Cognitive Decline / Depression Onset Profiles after ICH. Association between ICH volume and patient subgroups identified by specific profiles in cognitive and mood symptoms after ICH. Top panel: box and whishers plot presenting median (horizontal line within box), inter-quartile (box margins) and minim / maximum (upper and lower whishers) values for hematoma volume in each profile subgroup. Bottom Panel: area graph presenting the relationship between increasing hematoma volume and individual participants’ probability of expressing a specific profile. The solid vertical line indicates the median ICH volume value among study participants, while the shaded area encompasses the 95% confidence interval. Abbreviations: CSVD = Cerebral Small Vessel Disease, ICH = Intracerebral Hemorrhage

We created univariable and multivariable models exploring predictors of different profiles for depression and dementia incidence after ICH. In univariable analyses (Table 2) we found that history of pre-ICH depression, hematoma volume, disability at time of discharge from index ICH hospitalization, presence of intraventricular hemorrhage, CSVD severity on MRI, prevalence of CAA-related hemorrhages and APOE genotype differed significantly across profiles. We present in Table 3 results of multivariable analyses with Profile I (low depression and dementia risk) as reference, since it represents the most favorable clinical outcome. We also provide full results for all comparisons across profiles in Supplemental Table I. We found that history of pre-ICH depression, larger hematoma volume, presence of intraventricular hemorrhage, and more severe disability at time of index ICH hospitalization all independently predicted expression of Profile II (early depression and dementia risk). Greater CSVD severity on MRI, CAA hemorrhage etiology and APOE variant ε4 all independently predicted expression of Profile III (late depression and dementia risk). History of pre-ICH depression was the only independent predictor for expression of Profile IV (high depression and low dementia risk). We provide a graphical representation of the relationship between CSVD severity and cognitive decline and depression onset profiles in Fig. 3. We also illustrate the relationship between hematoma volume and symptoms’ profiles after ICH in Fig. 4.

**Table 2:** Univariable Analyses of Predictors for Cognitive Decline and Depression Onset Profiles after ICH

Variable	Depression / Dementia Profiles after ICH				p
	Profile I Low Depression and Dementia Risk	Profile II Early Depression and Dementia	Profile III Late Depression and Dementia	Profile IV High Depression with Low Dementia Risk	
**No. of individuals**	**75**	**189**	**387**	**133**	–
Pre-ICH Depression	12 (13)	37 (20)	58 (15)	28 (21)	**0.003**
ICH Volume (median, IQR)	18.1 (6.3–28.8)	26.5 (9.6–33.2)	20.2 (7.2–26.4)	17.8 (6.4–27.5)	**< 0.001**
Intraventricular Hemorrhage	19 (25)	62 (33)	89 (23)	34 (26)	**0.035**
Discharge mRS (median, IQR)	4 (3–4)	4 (4–5)	4 (3–5)	4 (3–5)	0.075
CSVD MRI Score (median, IQR)	2 (1–2)	2 (1–3)	3 (2–3)	2 (1–3)	**0.041**
CAA-related ICH	33 (44)	93 (49)	219 (57)	55 (41)	**0.002**
APOE ε2 (≥ 1 copy)	8 (11)	30 (16)	55 (14)	17 (13)	0.085
APOE ε4 (≥ 1 copy)	13 (17)	32 (17)	97 (25)	23 (17)	**0.012**

*P*-values reported for univariable comparisons across patient subgroups defined by specific profiles in cognitive and mood symptoms after ICH. We report results for variables with *p* < 0.20 in univariable analyses

Abbreviations: *CAA* Cerebral Amyloid Angiopathy, *CSVD* Cerebral Small Vessel Disease, *ICH* Intracerebral Hemorrhage, *IQR* Inter-quartile Range, *mRS* modified Rankin Scale

**Table 3:** Multivariable Analyses of Predictors for Cognitive Decline and Depression Onset Profiles after ICH

Predictor Variables	Cognitive Decline and Depression Onset Profiles							
	Profile I Low Depression and Dementia Risk		Profile II Early Depression and Dementia		Profile III Late Depression and Dementia		Profile IV High Depression with Low Dementia Risk	
	RRR (95% CI)	p	RRR (95% CI)	p	RRR (95% CI)	p	RRR (95% CI)	p
Pre-ICH Depression	Ref.	–	1.83 (1.16–2.86)	**0.011**	0.89 (0.71–1.11)	0.31	2.14 (1.25–3.64)	**0.008**
ICH Volume (per 10 cc increase)	Ref.	–	1.38 (1.03–1.84)	**0.041**	1.52 (0.73–3.14)	0.28	0.75 (0.54–1.02)	0.098
Intraventricular Hemorrhage	Ref.	–	1.78 (1.07–2.95)	**0.031**	0.68 (0.38–1.20)	0.20	1.28 (0.69–2.35)	0.45
Discharge mRS (per 1 point increase)	Ref.	–	1.41 (1.09–1.82)	**0.009**	1.18 (0.94–1.47)	0.17	0.67 (0.44–1.01)	0.071
CSVD MRI Score (per 1 point increase)	Ref.	–	1.11 (0.85–1.44)	0.44	1.28 (1.05–1.55)	**0.021**	0.71 (0.40–1.25)	0.25
CAA-related ICH	Ref.	–	1.29 (0.95–1.73)	0.11	1.84 (1.21–2.79)	**0.007**	1.36 (0.93–1.98)	0.13
APOE ε2 (≥ 1 copy)	Ref.	–	1.28 (0.99–1.64)	0.063	1.37 (0.92–2.02)	0.13	0.88 (0.40–1.91)	0.75
APOE ε4 (≥ 1 copy)	Ref.	–	1.12 (0.84–1.48)	0.45	1.57 (1.07–2.29)	**0.028**	0.79 (0.37–1.65)	0.55

Results from multivariable logistic regression analyses of risk factors for participants’ assignment to specific profiles in cognitive and mood symptoms after ICH. Effect sizes (RRR) and p-values represent comparison for each group with the patient subset at low risk for both dementia and depression after ICH, which was selected as reference

Abbreviations: *95% CI* 95% Confidence Interval, *CAA* Cerebral Amyloid Angiopathy, *CSVD* Cerebral Small Vessel Disease, *ICH* Intracerebral Hemorrhage, *IQR* Inter-quartile Range, *mRS* modified Rankin Scale, *RRR* Relative Risk Ratio

## Discussion

We leveraged latent profile analysis to identify distinct profiles in cognitive decline and depression onset in a large cohort of hemorrhagic stroke survivors with extended follow-up. We identified four distinct profiles, i.e. low depression and dementia risk (Profile I), early onset depression and dementia (Profile II), late-onset depression and dementia (Profile III), and high depression with low dementia risk (Profile IV). We also demonstrated that established risk factors for cognitive decline and depression after ICH predicted individual participants’ likelihood of expressing each of these profiles. Patients experiencing larger hemorrhages associated with more severe disability were more likely to express Profile II (early-onset depression and dementia). In contrast, neuroimaging and genetic markers of CSVD predicted expression of Profile III (late-onset depression and dementia). Our findings advance our understanding of the biological mechanisms underlying cognitive and depressive symptoms at different time points in the natural history of ICH. These insights will inform future prevention and treatment studies for cognitive and psychiatric sequelae of hemorrhagic stroke. Future clinical trials, in particular, will benefit from incorporation of our approach in study design. If further validated, this methodology could also be employed at bedside to assist in prognostication of cognitive decline and depression risk after ICH.

Our analyses indicate that early-onset dementia and depression after ICH are associated with larger hematoma size, intraventricular hemorrhage, and more severe disability at time of stroke). We previously demonstrated that larger hemorrhages were specifically associated with risk of early dementia after ICH [19]. Taken together, available evidence supports the hypothesis that early onset of dementia and depression after ICH reflects acute mechanical disruption of cerebral networks responsible for cognitive and affective functions. Of note, previous studies clearly identified hematoma size as a key determinant of disability after ICH [32]. However, we found that disability at time of discharge from index ICH hospitalization was an independent predictor for early-onset dementia and depression, even after adjustment for hemorrhage volume. This finding likely reflects the impact of post-ICH functional impairment on social engagement and ability to participate in rehabilitation, which were previously shown to increase risk for dementia and depression among stroke survivors [33].

History of pre-ICH depression predicted expression of either Profile II (early-onset depression and dementia) or Profile IV (depression with lower dementia risk). These findings may reflect the combination of individual predisposition to developing depressive symptoms with the acute ICH event, resulting in greater disruption of affective functioning for these patients. In contrast, we found no association between depression history before ICH and late-onset of dementia and depression afterwards. These findings are consistent with studies of stroke-free individuals diagnosed with vascular depression, among whom prior history of depression earlier in life was relatively infrequent (compared to elderly individuals diagnosed with major depressive disorder) [34]. In agreement with the “vascular depression” hypothesis, [13, 35] our results suggest progression of underlying CSVD to primarily account for delayed onset of depression after ICH, rather than individuals’ lifelong propensity to develop depression (as determined by genetics and pre-stroke environmental exposures). Overall, our results support screening for pre-ICH depressive symptoms to inform counseling and subsequent care of ICH survivors.

We found that increasing CSVD severity (especially of the CAA subtype) was specifically predictive of late-onset depression and dementia. We previously demonstrated that CSVD-related neuroimaging markers on MRI (cerebral microbleeds, white matter hyperintensities, lacunes, cortical superficial siderosis, and enlarged perivascular spaces) are potent predictors of cognitive decline risk after ICH [27]. Specifically, greater CSVD severity was shown to specifically increase risk for post ICH dementia beyond the first 6–12 months after the acute hemorrhage [19, 27]. CSVD-related MRI markers are also strongly associated with higher incidence of depressive symptoms in the general population, [4, 8] reflecting chronic accumulations of microvascular damage to cerebral structures involved in mood regulation. Because they often already harbor severe underlying CSVD, [27] ICH survivors are at very high risk for delayed neurocognitive and neuropsychiatric symptoms, regardless of the severity of the acute hemorrhage. Arresting or reversing the progression of CSVD after ICH may therefore contribute to staving off delayed onset of highly disabling cognitive and mood symptoms, which represented the most common outcome for ICH survivors in our study.

Our study has some limitations. We obtained information on cognitive performance and affective symptoms during follow-up primarily via telephone based evaluations, rather than during in-person interviews. The use of phone-based instruments might have resulted in limited sensitivity for milder cognitive and depressive symptoms among participants. In particular, the instrument used in our study to assess depressive symptoms after ICH (GDS-4), is a brief self-report measure. This may have resulted in systematic underestimation of depression incidence in our study. However, we previously demonstrated that our phone-based evaluation has excellent sensitivity and specificity for diagnosis of dementia and depression after ICH when compared to in-person evaluation conducted by specialists in neurology or psychiatry [3, 19]. Furthermore, in the present study application of LPA to phone-based assessments of cognitive and depressive symptoms resulted in highly specific outcome profiles of immediate clinical relevance, each displaying unique associations with clinical, genetic and neuroimaging data - thus further validating our approach. Our findings therefore support the hypothesis that even brief self-report measures may be leveraged using LPA to inform bedside tools for prognostication of depression risk after stroke. Finally, the cohort for this study includes only individuals enrolled at a tertiary care center with expertise in ICH care, potentially limiting generalizability to all hemorrhage survivors. Future studies should expand upon our findings in different health-care settings, and include a more diverse population of ICH survivors. Our approach also has several strengths. We used a standardized longitudinal follow-up methodology to collect extensive clinical, genetic, neuroimaging and outcome data on a large group of ICH survivors. As a result, we were able to consistently apply a robust, validated bioinformatics approach in a novel manner and create detailed models of cognitive and affective functions after ICH. We also ensured low rates of loss to follow up in a vulnerable population at high risk for recurrent stroke, dementia, and depression.

In summary, we provide evidence of distinct profiles for cognitive and depressive symptoms in a large cohort of consecutive ICH survivors. Each profile represents a distinct clinical-biological phenotype, with highly specific association with medical history, neuroimaging and genetic information. As a result, we were able to explore differences in biological mechanisms underlying development of depression and dementia at different time points after hemorrhagic stroke. If further validated, this innovative approach could be leveraged to guide prognostication and clinical care for ICH survivors. Future studies of ICH may also benefit from its application, whether in investigating neurocognitive and neuropsychiatric outcomes or selecting more appropriate patient subgroups for interventional trials. Application to other hemorrhagic and ischemic stroke subtypes is also warranted, and may result in additional, disease-specific biological insights into cognitive decline and depression onset among survivors.

## Supplementary Information

**Additional file 1: Supplemental Methods. Supplementary Table I.** Multivariable Analyses of Predictors for Cognitive Decline and Depression Onset Profiles after ICH.

## Data Availability

The datasets used and/or analyzed during the current study are available from the corresponding author on reasonable request.

## Abbreviations

ICH: Intracerebral Hemorrhage
LPA: Latent Profile Analysis
CSVD: Cerebral small vessel disease
MRI: Magnetic Resonance Imaging
CT: Computer tomography imaging
MGH-ICH: Study of ICH survivors conducted at Massachusetts General Hospital
mRS: Modified Rankin Scale
ADLS: Katz and Lawton questionnaires for Activities of Daily Living
IADLS: Instrumental Activities of Daily Living
GDS-4: 4-item version of the Geriatric Depression Scale
TICS-m: Modified Telephone Interview for Cognitive Status
IQ CODE: Informant Questionnaire on Cognitive Decline in the Elderly
VIF: Variance Inflation Factors
FDR: False Discovery Rate
BIC: Bayesian Information Criterion
IVH: Intraventricular Hemorrhage
CMBs: Cerebral microbleeds
EPVS: Expanded Peri-Vascular Spaces
WMH: White Matter Hyperintensities
CSS: Cortical Superficial Siderosis
HTNA: Hypertensive Arteriopathy
CAA: Cerebral Amyloid Angiopathy
