# Supplementary material for: Latent profile analysis of cognitive decline and depressive symptoms after intracerebral hemorrhage
Source: BMC Neurol. 2021 Dec 10;21:481. doi: 10.1186/s12883-021-02508-x (PMC8662844; doi:10.1186/s12883-021-02508-x)
Supplement: Supplementary file 1 — Additional file 1: Supplemental Methods. Supplementary Table I. Multivariable Analyses of Predictors for Cognitive Decline and Depression Onset Profiles after ICH. [file 12883_2021_2508_MOESM1_ESM.docx]

**Latent Profile Analysis of Cognitive Decline and Depressive Symptoms**

**after Intracerebral Hemorrhage**

Keins S. et al.

**SUPPLEMENTAL MATERIAL**

**Supplemental Methods**

*MRI Data Collection and Analysis*

MRI Images were obtained using a 1.5 or 3.0 Tesla MR scanner (GE Sigma) that included T1 weighted, T2-weighted, fluid attenuated inversion recovery (FLAIR), diffusion weighted images (DWI) with apparent diffusion coefficient map, and T2*-weighted gradient-recalled echo (echo time 750/50ms, 5mm slice thickness, 1mm inter-slice gap).(1, 2) For a subset of patients, blood-sensitive MRI sequences included susceptibility-weighted images (SWI). In the infrequent scenario of a participant having both T2*-GRE and SWI available, we reviewed the SWI. MRI-based markers of CSVD were rated according to the Standards for Reporting Vascular Changes on Neuroimaging (STRIVE) consensus criteria.(3) We defined WMH as a hyperintense signal abnormality on FLAIR, without evidence of cavitation. WMH severity was evaluated in axial FLAIR sequences in deep (range 0-3) and periventricular regions (range 0-3) using the Fazekas scale.^35^ Lacunes were defined as small, round or ovoid, subcortical fluid-filled cavity of between 3 and 15 mm in diameter, that were hypointense on T1 imaging with corresponding hyperintense signal on FLAIR.(2) To prevent the incorrect classification of CMB as lacunes, GRE/SWI sequences were also reviewed. Lacunes were classified according to anatomical location as lobar and non-lobar.(2) We defined CMB as round or ovoid hypointense lesions less than 10 mm in diameter on axial blood-sensitive sequences, lacking signal hyperintensity on T1-weighted or T2-weighted sequences, and distinct from leptomeningeal hemosiderosis or vascular flow voids. CMB location and number were evaluated following consensus criteria as previously described.(4) EPVS were defined as small, linear or round structures of cerebrospinal fluid (CSF) intensity, less than 3 mm in diameter, that follow the path of perforating cerebral vessels. EPVS were visually rated on axial T2-weighted MRI images, in the basal ganglia (BG) and centrum semiovale (CSO) utilizing a validated 4-point visual rating scale (0 = no EPVS, 1 < 10 EPVS, 2 = 11–20 EPVS, 3 = 21–40 EPVS, and 4 = > 40 EPVS).(5) We defined cSS as a distinct, curvilinear hypointensity on blood-sensitive MRI sequences (GRE/SWI) over the surface of the cerebral cortex and classified it as focal (restricted to three or less sulci) and disseminated (diffuse to more than 3 sulci).(6) All MRI scans were independently manually reviewed by two clinicians (with MD degrees), who completed a three-months training course including supervised reading of 20 training MRI scans and graded adjudication of 10 MRI scans. All scans were subsequently reviewed, and grading confirmed by a board-certified senior neurologist with at least five years of expertise in clinical practice. Disagreements among junior graders and/or between junior and senior graders were referred for final adjudication to a panel of three board-certified neuroradiologists with at least 10 years of expertise in clinical practice. The median intra-class correlation among graders for MRI scans was 0.93 (range 0.92 - 0.95), and therefore deemed excellent.

*ICH Etiological Classification*

All ICH survivors were classified based on the location of ICH and CMBs, and presence of cSS as HTNA-related ICH, CAA-related ICH and mixed-ICH.(1) HTNA-related ICH patients had strictly deep (when located in the BG, thalamus, brainstem and cerebellum) ICH with or without exclusively deep located CMBs (lobar CMBs and cSS not allowed). CAA-related ICH patients had lobar hemorrhages involving the cerebral cortex and the underlying white matter with or without exclusively lobar located CMBs and cSS (deep located CMBs not allowed), fulfilling modified Boston criteria for probable and possible CAA.(7) Mixed-ICH patients met one of the following criteria: 1) lobar ICH and ≥1 deep CMBs, 2) deep ICH and ≥1 lobar CMBs or presence of cSS, or 3) deep and lobar ICHs with or without CMBs in any location. (1)

**Supplementary Table I: Multivariable Analyses of Predictors for Cognitive Decline and Depression Onset Profiles after ICH**

| **Predictor**  **Variables** | **Cognitive Decline and Depression Onset Profiles’ Comparisons** | | | | | |
| --- | --- | --- | --- | --- | --- | --- |
|  | **Profile I** (ref)  vs.  **Profile II** | **Profile I** (ref)  vs.  **Profile III** | **Profile I** (ref)  vs.  **Profile IV** | **Profile II** (ref)  vs.  **Profile III** | **Profile II** (ref)  vs.  **Profile IV** | **Profile III** (ref)  vs.  **Profile IV** |
|  | **RRR**  **(95% CI)** | **RRR**  **(95% CI)** | **RRR**  **(95% CI)** | **RRR**  **(95% CI)** | **RRR**  **(95% CI)** | **RRR**  **(95% CI)** |
| Pre-ICH  Depression | **1.83**  **(1.16 - 2.86)** | 0.89  (0.71 - 1.11) | **2.14**  **(1.25 - 3.64)** | **0.62**  **(0.43 - 0.87)** | 0.80  (0.46 - 1.38) | **1.89**  **(1.19 - 3.01)** |
| ICH Volume  (per 10 cc incr.) | **1.38**  **(1.03 - 1.84)** | 1.52  (0.73 - 3.14) | 0.75  (0.54 - 1.02) | **0.78**  **(0.64 - 0.94)** | **0.82**  **(0.70 - 0.95)** | 1.28  (0.82 - 1.97) |
| Intraventricular  Hemorrhage | **1.78**  **(1.07 - 2.95)** | 0.68  (0.38 - 1.20) | 1.28  (0.69 - 2.35) | **0.69**  **(0.52 - 0.90)** | **0.62**  **(0.42 - 0.91)** | 1.18  (0.80 - 1.73) |
| Discharge mRS  (per 1 point incr.) | **1.41**  **(1.09 - 1.82)** | 1.18  (0.94 - 1.47) | 0.67  (0.44 - 1.01) | **0.77**  **(0.62 - 0.94)** | 0.92  (0.58 - 1.44) | 0.84  (0.60 - 1.17) |
| CSVD MRI Score  (per 1 point incr.) | 1.11  (0.85 - 1.44) | **1.28**  **(1.05 - 1.55)** | 0.71  (0.40 - 1.25) | **1.31**  **(1.04 - 1.63)** | 0.84  (0.53 - 1.32) | **0.81**  **(0.69 - 0.94)** |
| CAA-related ICH | 1.29  (0.95 - 1.73) | **1.84**  **(1.21 - 2.79)** | 1.36  (0.93 - 1.98) | **1.65**  **(1.14 - 2.38)** | 1.28  (0.88 - 1.84) | **0.74**  **(0.58 - 0.93)** |
| APOE ε2  (≥ 1 copy) | 1.28  (0.99 -1.64) | 1.37  (0.92 - 2.02) | 0.88  (0.40 - 1.91) | 1.44  (0.99 - 2.09) | 0.84  (0.68 - 1.02) | **0.88**  **(0.78 - 0.98)** |
| APOE ε4  (≥ 1 copy) | 1.12  (0.84 - 1.48) | **1.57**  **(1.07 - 2.29)** | 0.79  (0.37 - 1.65) | **1.48**  **(1.08 - 2.02)** | 1.29  (0.60 - 2.74) | **0.74**  **(0.58 - 0.92)** |

Results from multivariable logistic regression analyses of risk factors for participants’ assignment to specific profiles in cognitive and mood symptoms after ICH. Effect sizes (RRR) represent comparison for two patients’ groups, based on reported profiles. Bolded results achieved statistical significance after adjustment for multiple testing.

Profile legend as follows:

Profile I: low depression and low dementia risk

Profile II: early depression and dementia

Profile III: late depression and dementia

Profile IV: high depression and low dementia risk

Abbreviations: 95% CI = 95% Confidence Interval, CAA = Cerebral Amyloid Angiopathy, CSVD = Cerebral Small Vessel Disease, ICH = Intracerebral Hemorrhage, IQR = Inter-quartile Range, mRS = modified Rankin Scale, RRR = Relative Risk Ratio.

**REFERENCES**

1. Pasi M, Charidimou A, Boulouis G, Auriel E, Ayres A, Schwab KM, et al. Mixed-location cerebral hemorrhage/microbleeds: Underlying microangiopathy and recurrence risk. Neurology. 2018;90(2):e119-e26.

2. Pasi M, Boulouis G, Fotiadis P, Auriel E, Charidimou A, Haley K, et al. Distribution of lacunes in cerebral amyloid angiopathy and hypertensive small vessel disease. Neurology. 2017.

3. Wardlaw JM, Smith EE, Biessels GJ, Cordonnier C, Fazekas F, Frayne R, et al. Neuroimaging standards for research into small vessel disease and its contribution to ageing and neurodegeneration. Lancet neurology. 2013;12(8):822-38.

4. Greenberg SM, Vernooij MW, Cordonnier C, Viswanathan A, Al-Shahi Salman R, Warach S, et al. Cerebral microbleeds: a guide to detection and interpretation. Lancet neurology. 2009;8(2):165-74.

5. Charidimou A, Boulouis G, Pasi M, Auriel E, van Etten ES, Haley K, et al. MRI-visible perivascular spaces in cerebral amyloid angiopathy and hypertensive arteriopathy. Neurology. 2017;88(12):1157-64.

6. Charidimou A, Boulouis G, Fotiadis P, Xiong L, Ayres AM, Schwab KM, et al. Acute convexity subarachnoid haemorrhage and cortical superficial siderosis in probable cerebral amyloid angiopathy without lobar haemorrhage. Journal of neurology, neurosurgery, and psychiatry. 2018;89(4):397-403.

7. Linn J, Halpin A, Demaerel P, Ruhland J, Giese AD, Dichgans M, et al. Prevalence of superficial siderosis in patients with cerebral amyloid angiopathy. Neurology. 2010;74(17):1346-50.
